# Effects of Dietary Tannic Acid and Tea Polyphenol Supplementation on Rumen Fermentation, Methane Emissions, Milk Protein Synthesis and Microbiota in Cows

**DOI:** 10.3390/microorganisms13081848

**Published:** 2025-08-07

**Authors:** Rong Zhao, Jiajin Sun, Yitong Lin, Haichao Yan, Shiyue Zhang, Wenjie Huo, Lei Chen, Qiang Liu, Cong Wang, Gang Guo

**Affiliations:** College of Animal Science, Shanxi Agricultural University, Jinzhong 030800, China; 19835144089@163.com (R.Z.); ss20000729@163.com (J.S.); 18242527502@163.com (Y.L.); 16634236243@163.com (H.Y.); zsy15735850558@163.com (S.Z.); huohuo-1982@163.com (W.H.); cl1016zj@126.com (L.C.); liuqiangabc@163.com (Q.L.); wangdx0321@163.com (C.W.)

**Keywords:** tannin, tea polyphenols, methane emissions, milk quality, rumen fermentation

## Abstract

To develop sustainable strategies for mitigating ruminal methanogenesis and improving nitrogen efficiency in dairy systems, this study investigated how low-dose tannic acid (T), tea polyphenols (TP), and their combination (T+TP; 50:50) modulate rumen microbiota and function. A sample of Holstein cows were given four dietary treatments: (1) control (basal diet); (2) T (basal diet + 0.4% DM tannic acid); (3) TP (basal diet + 0.4% DM tea polyphenols); and (4) T+TP (basal diet + 0.2% DM tannic acid + 0.2% DM tea polyphenols). We comprehensively analyzed rumen fermentation, methane production, nutrient digestibility, milk parameters, and microbiota dynamics. Compared with the control group, all diets supplemented with additives significantly reduced enteric methane production (13.68% for T, 11.40% for TP, and 10.89% for T+TP) and significantly increased milk protein yield. The crude protein digestibility significantly increased in the T group versus control. The results did not impair rumen health or fiber digestion. Critically, microbiota analysis revealed treatment-specific modulation: the T group showed decreased *Ruminococcus flavefaciens* abundance, while all tannin treatments reduced abundances of *Ruminococcus albus* and total methanogens. These microbial shifts corresponded with functional outcomes—most notably, the T+TP synergy drove the largest reductions in rumen ammonia-N (34.5%) and milk urea nitrogen (21.1%). Supplementation at 0.4% DM, particularly the T+TP combination, effectively enhances nitrogen efficiency and milk protein synthesis while reducing methane emissions through targeted modulation of key rumen microbiota populations, suggesting potential sustainability benefits linked to altered rumen fermentation.

## 1. Introduction

The rising atmospheric concentration of greenhouse gases represents a critical global environmental challenge [1]. Mitigating GHG emissions, particularly methane (CH_4_), has become an urgent international priority. Livestock production contributes significantly to anthropogenic emissions, accounting for 14.5% of the global total, with enteric CH_4_ comprising 39% of this sector’s emissions [2]. Within ruminant livestock, CH_4_ is primarily generated through microbial fermentation of carbohydrates in the rumen [3]. Projected increases in global demand for ruminant-derived products (meat, milk) will necessitate herd expansion, inevitably leading to higher absolute methane (CH_4_) emissions. While system intensification can reduce emissions per unit of product, these efficiency gains may not be sufficient to achieve the necessary absolute reductions in methane output. Consequently, mitigating the associated environmental impacts remains a significant challenge. These emissions impose a dual burden: they exacerbate climate change and represent substantial energy loss, estimated at 2–12% of gross feed energy intake, equivalent to ~6% of ingested energy or 12% of digestible energy in cattle [4,5]. Consequently, developing effective feed additives to suppress ruminal methanogenesis is essential for achieving sustainable livestock production and enhancing feed conversion efficiency.

The methanogenic potential of the rumen microbiota is significantly influenced by its bacterial structure. In the strictly anaerobic rumen environment [6], key microbes (Protozoa, *R. albus*, *R. flavefaciens*, and *R. amylophilus*) ferment dietary fiber and starch into volatile fatty acids (VFAs). This fermentation process generates substantial hydrogen gas. Methanogens primarily utilize this hydrogen to reduce carbon dioxide into methane [7]. When methanogens are highly active, excessive hydrogen consumption can alter the balance of hydrogen partial pressure and shift fermentation patterns. This typically redirects the hydrogen sink toward methane production rather than propionate synthesis, increasing the proportion of acetate and butyrate. Consequently, hydrogen is diverted from propionate synthesis to methane generation, impacting microbial nitrogen metabolism. Since propionate synthesis requires hydrogen, its positive correlation with nitrogen efficiency indicates that enhancing hydrogen utilization can boost nitrogen assimilation. Meanwhile, *R. amylophilus* produces LA (lactic acid), while *Prevotella* uses it to generate propionate through the succinate pathway. Reducing methane emissions may thus elevate propionate levels, enhancing host energy and increasing milk protein content. Therefore, developing additives that leverage these microbial functional dynamics and interaction mechanisms is crucial for effective methane reduction and improved animal production efficiency.

In recent years, numerous plant extracts rich in secondary metabolites (including terpenoids, neem extracts, saponins, and flavonoids) have been evaluated for their potential to reduce ruminal methane (CH_4_) production. Studies consistently report the ability of these additives to inhibit CH_4_ generation [8,9,10,11,12]. Among these, tannins—natural polyphenolic compounds found in plants such as shrubs, legumes, and cereals—are categorized primarily into hydrolysable tannins (HT) and condensed tannins (CT) based on structural differences [13], and have garnered significant attention in methane mitigation research. Existing research clearly demonstrates that hydrolysable tannins (HT) effectively inhibit the activity of ruminal methanogens, thereby reducing methane production [14]. By binding to macromolecules, CT reduces the degradation of proteins and carbohydrates, thereby significantly enhancing its functional efficacy [15]. Battelli et al. [16] demonstrated that supplementing 8% quebracho CT reduced cumulative CH_4_ production by 17.5% during 48 h fermentation, significantly exceeding the 10.4% reduction achieved with chestnut HT. These findings underscore CT potential as a targeted methane inhibitor in ruminant nutrition strategies. However, tea polyphenols (TP)—a potent source of CT rich in epigallocatechin gallate [17]—although well-documented for their antimicrobial and antioxidant efficacy in monogastric animals, remain underexplored in ruminants. Notably, emerging evidence suggests synergistic potential between HT and CT [18,19], yet the combinatorial effects and underlying mechanisms are poorly characterized. The role of TP in dairy production systems is virtually unstudied. Beyond methane reduction, tannins play a crucial role in modulating rumen microbial function, promoting microbial protein synthesis and nitrogen utilization efficiency [20], while also demonstrating potential as antibiotic alternatives [21]. Specifically, HT has been reported to reduce ruminal excessive protein degradation and methane generation by inhibiting specific rumen microbial functions. Simultaneously, it increases protein digestion and absorption in the small intestine, ultimately enhancing nitrogen (N) utilization efficiency in ruminants [22,23].

Based on these findings, we hypothesize that the individual and combined supplementation of two tannins at equal doses can effectively reduce enteric CH_4_ emissions by altering the rumen microbiota. This study aimed to suppress methanogens and *Ruminococcus albus* while preserving key fibrolytic communities, explaining the observed maintenance of fiber digestibility alongside methane mitigation, without negatively impacting animal production. This study aims to investigate the effects of individual and combined tannin supplementation on rumen fermentation (fermentation parameters, CH_4_ production, and microbial community structure), nutrient digestibility, lactation performance, and blood biochemical parameters in dairy cows.

## 2. Materials and Methods

### 2.1. Experimental Design and Feeding Management

All experimental procedures involving animal care and management were authorized by the Institutional Animal Care and Use Committee of Shanxi Agricultural University located in Taigu District, Jinzhong City, Shanxi Province, China (Approval code. SXAU-EAW-2022C.RD.010025174. Approval Date: 25 November 2022). The experimental pasture is located at Shanxi Wangxiangyuan Animal Husbandry Co., Ltd., situated west of Gao Village, Beige Town, Xiaodian District, Taiyuan, Shanxi Province, China. The animal study was performed according to the Regulation on the Administration of Laboratory Animals (2017 Revision) promulgated by the State Council.

Twenty-four dairy cows with similar parity (second), lactation days (130 ± 9 d), milk yield (36.0 ± 1.1 kg), body weight (635 ± 8 kg), and body condition score (2.96 ± 0.08) were randomly assigned into four groups, with six cows per group. The experimental treatments were as follows: (1) group one was fed a basal diet (C, control); (2) group two was fed a basal diet plus 0.4% DM tannic acid (T, tannin); (3) group four was fed a basal diet plus 0.4% DM tea polyphenols (TP, tea polyphenols); (4) group three was fed a basal diet plus 0.2% DM tannic acid and 0.2% DM tea polyphenols (T+TP, tannin+tea polyphenols). The cows were given a 10-day pre-experimental period and a 40-day formal experimental period. During the pre-experimental period, the cows’ feeding, rumination, feces, and body condition were observed. The cows’ TMR (Total Mixed Diet) was formulated according to NRC [24]. The diet composition and analysis levels of the experimental diets are shown in Table 1.

### 2.2. Milk Yield Recording and Milk Sample Collection

On days 10, 20, and 30 of the formal period, milk samples were collected and the daily milk yield of the cows was recorded to calculate the average milk yield over 10 days. For each sampling, a mixture of morning, afternoon, and evening milk (in a ratio of 4:3:3) was collected, totaling 50 mL. Potassium dichromate was added as a preservative, and the samples were stored at a low temperature for subsequent milk component analysis.

### 2.3. Sample Collection of Blood, Ruminal Fluid, and Feces

Commencing on day 31 of the experimental period, fecal samples were collected rectally from each cow three times daily (morning, noon, evening) for 5 consecutive days. Samples were immediately mixed with a 25% aqueous tartaric acid solution (10% *w*/*v*) and stored at −20 °C. On the morning of day 40 prior to feeding, blood was collected from the tail vein using 10 mL vacuum tubes. Samples were centrifuged at 1500× *g* for 10 min to obtain serum. The supernatant serum was aliquoted into 2 mL microcentrifuge tubes and stored at −20 °C. On day 40 at 3 h post-morning feeding, rumen fluid (about 100 mL) was collected using an ororuminal probe equipped with a metal strainer and manual pump with glass container. Fluid was filtered through four layers of sterile cheesecloth. pH was measured immediately. Filtered rumen fluid was then aliquoted into 10 mL sterile tubes and stored at −80 °C for subsequent analysis.

### 2.4. Methane Gas Collection

From day 34 of the experiment, gas was collected from each cow for two consecutive days. A respiratory mask (Beijing Zhengfang Xingda Technology Development Co., Ltd., Beijing, China) gas collection device was used [25]. Gas collection was conducted four times daily: at 6:00 (one hour before feeding), 12:00 (representing the daytime rest period), 20:00 (one hour after feeding), and 24:00 (representing the nighttime rest period). Each time, the time taken to fill the gas bag (97 cm × 63 cm × 63 cm ≈ 385 L) was recorded. A gas analyzer (Beijing Zhuoan Hengrui Technology Co., Ltd., Beijing, China) was used to determine the concentration of each gas component in the bag, and CH_4_ emissions were calculated [26].

### 2.5. Analysis of Rumen Fermentation Characteristics and Serum Indexes

Ruminal fluid samples were first thawed. Then, 5 mL of the sample was mixed with 1 mL of 25% metaphosphate and centrifuged at 15,000 rpm and 4 °C for 10 min. The supernatant was used for VFA analysis via gas chromatograph (GC122 gas chromatograph, Shanghai Precision Scientific Instrument Co., Ltd., Shanghai, China) with a capillary column [27]. The chromatograph settings were as follows: carrier gas N_2_, split ratio 40:1, injection volume 0.6 µL, detector FID with a temperature of 230 °C, H_2_ flow 40 mL/min, air flow 450 mL/min, make-up gas flow 45 mL/min. The column oven temperature rose from 120 to 180 °C at 10 °C/min. Standard solutions were made by mixing specific volumes of VFAs in volumetric flasks and diluting with distilled water.

Following thawing on ice, serum samples were centrifuged at 3000× *g* for 10 min at 4 °C. Biochemical parameters in the supernatant were quantified using a clinical chemistry analyzer (BS-360S, Shenzhen Mindray Bio-Medical Electronics Co., Ltd., Shenzhen, China) and included the following: Triglycerides (TG), Total cholesterol (TC), Urea nitrogen (UN), Creatinine (CRE), Aspartate aminotransferase (AST), Alanine aminotransferase (ALT), Albumin (ALB), Gamma-glutamyl transferase (GGT), Total protein (TP), and Globulin (GLB). Rumen ammonia nitrogen (NH_3_-N) was quantified by phenol-hypochlorite colorimetry [28] using a spectrophotometer (UV-1800, Shanghai Meipuda Instrument Co., Ltd., Shanghai, China). Microbial crude protein (MCP) in rumen fluid was determined via Coomassie Brilliant Blue colorimetric assay with absorbance measured at 595 nm on the same instrument [29].

### 2.6. Milk Yield Recording and DHI Analysis

Milk yield was automatically recorded daily at the milking parlor, with production data from the preceding day extracted on experimental days 10, 21, and 31. Composite milk samples were submitted to the Taiyuan DHI Testing Center (Taiyuan, China) for standard milk composition analysis, including milk yield, fat-corrected milk (FCM), milk protein yield, milk fat, milk protein, lactose, and solids-not-fat (SNF). Analyses were performed using a Fossomatic™ 5000 series instrument (FOSS Analytical A/S, Hillerød, Denmark).

### 2.7. Analysis of Apparent Digestibility

Fresh feed, feed remnants, and feces were analyzed for crude protein (CP), dry matter (DM), ether extract (EE), and organic matter (OM) using AOAC methods [30]. Neutral detergent fiber (NDF) and acid detergent fiber (ADF) were determined via an Ankom fiber analyzer. Apparent digestibility (AD) was calculated as follows: AD (%) = [(intake − fecal content)/intake] × 100.

### 2.8. Microbial DNA Extraction and Quantification

Ruminal microbial DNA was extracted following the method of Edrington TS [31]. qPCR was used to quantify the relative abundance of 10 bacteria in the incubation fluid, with primers listed in Table 2. To validate amplification efficiency for each qPCR assay, known quantities of the target species template were quantified. Standard curves were subsequently constructed. All assays demonstrated high linearity between Ct values and template dilution, with strong correlation coefficients (R^2^). Details are in the supporting information. Real-time PCR was performed on an Applied Biosystems Step One Plus system (USA). The 20 μL reaction mixture included 10 μL of SYBR Premix TaqTM (TaKaRa Biotechnology Co., Ltd., Beijing, China), 7.0 μL of ddH_2_O, 0.8 μL of each primer (0.2 μmol/L), 0.4 μL of ROX Reference Dye (50×), and 1 μL of template DNA. The Ct value for each sample was recorded. The PCR program comprised initial denaturation (50 °C for 2 min, 95 °C for 2 min) and 40 cycles of amplification (95 °C for 15 s, 60 °C for 1 min) [32,33]. To validate amplification efficiency for each qPCR assay, known quantities of target species template were quantified. Standard curves were subsequently constructed. All assays demonstrated high linearity between Ct values and template dilution, with strong correlation coefficients (R^2^). See Supplementary Figure S1.

### 2.9. Statistical Analysis

Experimental data was analyzed using the single-factor (ANOVA) module of the SPSS 27 software, and Duncan’s method was used for multiple comparisons. A Duncan test was used to assess the differences among the four groups. Results are expressed as mean ± standard deviations. The significant difference was defined as *p* < 0.05, and 0.05 < *p* < 0.10 was considered as a tendency.

## 3. Results

### 3.1. Effect of Tannic Acid and Tea Polyphenol Dietary Supplementation on Rumen Fermentation in Dairy Cows

As indicated in Table 3, the addition of tannins significantly (*p* < 0.05) increased the pH of the dairy cows’ rumen, but there were no significant (*p* > 0.05) differences among the T, T+TP, and TP groups. Compared with the control group (C), the groups supplemented with tannins had significantly (*p* < 0.05) lower levels of NH_3_-N and MCP, but no significant (*p* > 0.05) differences were observed among the three groups. The propionate content in the T, T+TP, and TP groups was significantly (*p* < 0.05) higher than that in the C group, with the T group showing a significantly (*p* < 0.05) higher propionate content than the TP group, and no significant (*p* > 0.05) difference between the T group and the T+TP group. The acetate to propionate ratio in the T, T+TP, and TP groups was significantly (*p* < 0.05) lower than that in the C group, with no significant (*p* > 0.05) differences among the three groups. The methane production in the groups treated with tannins was significantly (*p* < 0.05) lower than that in the C group, but there were no significant (*p* > 0.05) differences among the three treatment groups.

### 3.2. Effect of Tannic Acid and Tea Polyphenol Dietary Supplementation on Nutrient Apparent Digestibility of Dairy Cows

Table 4 indicates that there is a trend towards increased apparent digestibility of dry matter (DM) and organic matter (OM) in the T, T+TP, and TP groups compared to the C group (0.1 > *p* > 0.05). The apparent digestibility of crude protein (CP) in the T group was significantly (*p* < 0.05) higher than in the C group, while the apparent digestibility of CP in the T+TP and TP groups was intermediate between the T and C groups, with no significant (*p* > 0.05) differences between them. There were no significant (*p* > 0.05) changes in the apparent digestibility of ether extract (EE), acid detergent fiber (ADF), and neutral detergent fiber (NDF) among the four groups. Notably, EE and NDF trended downward across supplemented diets versus control, except ADF.

### 3.3. Effect of Tannic Acid and Tea Polyphenol Dietary Supplementation on Blood Indicators in Dairy Cows

Table 5 shows that the plasma urea nitrogen content in the T, T+TP, and TP groups was significantly (*p* < 0.05) lower than in the C group, with no significant (*p* > 0.05) differences among the three groups. Compared to the control group, there was a trend towards increased serum albumin and total protein content in the groups treated with tannins in the dairy cow diet, while there were no significant (*p* > 0.05) changes in triglycerides, total cholesterol, creatinine, aspartate aminotransferase, alanine aminotransferase, or globulin. Compared to the control group, there was a trend towards a decrease in glutamylphthalide aminotransferase in the TP groups.

### 3.4. Effect of Tannic Acid and Tea Polyphenol Dietary Supplementation on Ruminal Microbial Structure in Dairy Cows

**Figure 1:**
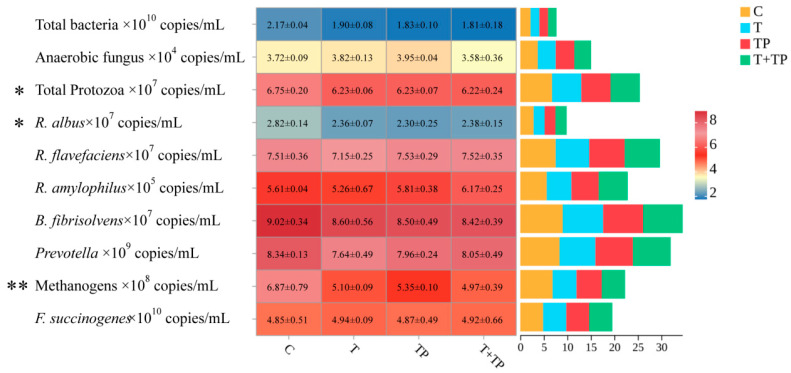
Effects of tannic acid and tea polyphenols on rumen microbiota structure in dairy cows. Note: C, without tannic acid and tea polyphenols; T, 0.4% tannic acid added; TP, 0.4% tea polyphenols added; T+TP, 0.2% tannic acid and 0.2% tea polyphenols added. * *p* < 0.05, ** *p* < 0.01.

Supplementation with tannic acid (T), tea polyphenols (TP), or their combination (T+TP) significantly modulated rumen microbial populations (Figure 1). The relative proportions of total bacteria tended to decrease in all treatment groups compared to control (*p* = 0.059), representing reductions of 12.4–16.6%. The relative proportions of methanogens were dramatically suppressed across treatments (*p* = 0.003), with reductions of 22.1–27.7%. Similarly, the relative proportions of *Ruminococcus albus* significantly declined in supplemented groups (*p* = 0.039), showing decreases of 16.3–18.4%. The relative proportions of protozoa were significantly reduced (*p* = 0.024), while the relative proportions of anaerobic fungi showed no significant changes (*p* = 0.281). *Ruminococcus flavefaciens* exhibited numerical reduction with T alone but no statistical significance (*p* = 0.426). Key fiber-degrading bacteria including *Fibrobacter succinogenes* (*p* = 0.904), *Butyrivibrio fibrisolvens* (*p* = 0.522), and *Prevotella* (*p* = 0.308) maintained stable populations across all treatments. *Ruminobacter amylophilus* showed a trend toward increase in T+TP but no significant group differences (*p* = 0.103). This specific microbial shift pattern—characterized by significant suppression of methanogens and *R. albus* without compromising major fibrolytic bacteria—supports the maintained fiber digestibility and methane reduction observed in the trial.

### 3.5. Effects of Tannic Acid and Tea Polyphenol Dietary Supplementation on Milk Yield and Components

Supplementation (Table 6) significantly increased milk protein percentage (*p* = 0.001) and decreased milk urea nitrogen (MUN, *p* = 0.022). Milk protein yield showed a tendency toward increase (*p* = 0.074), as did 4% fat-corrected milk yield (*p* = 0.089). No significant effects were observed on milk yield (*p* = 0.157), milk fat percentage (*p* = 0.464), lactose percentage (*p* = 0.673), non-fat milk solids (*p* = 0.437), total solids (*p* = 0.638), or somatic cell count (*p* = 0.153).

## 4. Discussion

The observed elevation in ruminal pH within this study can be attributed to several factors influencing the ruminal ecosystem, including salivary secretion, organic acid production, absorption/excretion rates, and dietary composition. Specifically, the addition of tannins likely contributed by binding dietary proteins, forming stable complexes resistant to microbial degradation within the typical ruminal pH range (5.5–7.5), and through their inherent antibacterial properties, thereby modulating rumen microbial fermentation [34]. This modulation is further evidenced by the significant changes in volatile fatty acid (VFA) profiles. The supplementation of 0.4% DM tannic acid, a tannic acid/tea polyphenol mixture (50:50), or tea polyphenols alone consistently increased propionic acid concentration and decreased the acetate-to-propionate ratio. While dietary polysaccharides are primarily fermented by rumen microbes to acetate, propionate, and butyrate (alongside CO_2_ and CH_4_), the impact of tannins on VFA molar ratios is often inconsistent across studies. For instance, Benchaar et al. [35] reported no effect on total VFA concentration or individual molar proportions with 0.64% DM quebracho tannin extract, whereas Dschaak et al. [36] observed a decrease in total VFA at a higher supplementation level (3% DM) of the same extract. These divergent findings regarding tannin effects on VFA profiles—encompassing total concentration and individual acid proportions—are likely influenced by key variables such as tannin dose, source (e.g., condensed vs. hydrolysable), interactions with dietary constituents, and the adaptation period of the rumen microbiota [37]. Supporting the dose-dependency, Carulla et al. [21] found no change in total VFA with 0.6% condensed tannins in sheep, but noted a shift towards propionate at the expense of acetate. In the present experiment, the significant alterations in propionate and the acetate:propionate ratio induced by all tannin-containing additives at 0.4% DM highlight their efficacy at this level. Notably, the combined tannic acid and tea polyphenol supplementation yielded superior results compared to tea polyphenols alone.

The synthesis of ruminal microbial protein (MCP) is intricately influenced by tannins, their supplementation level, and dietary composition, reflecting a complex interplay between tannins and the rumen microbiota. While tannins can inhibit microbial growth and enzyme activity, they may also promote MCP synthesis, with the net effect dependent on the balance of these opposing actions [38]. In this experiment, MCP content was significantly lower in tannin-supplemented groups compared to the control, with the combined tannic acid and tea polyphenol group exhibiting the lowest total bacterial abundance. Concurrently, tannins at 0.4% DM significantly reduced the abundance of methanogenic archaea and protozoa. This reduction in methanogens aligns with their ability to bind proteins or microbial enzymes and their symbiotic relationship with protozoa [3]. The decrease in protozoa is notable given their significant proteolytic role in degrading both rumen-degradable and undegradable protein (RDP and RUP) [17], particularly as crude protein content significantly increased in the experimental groups. The discrepancy between these results (significant reduction at 0.4% DM) and those of Yang K et al. (no effect at 0.65% DM, significant reduction at 2.6% DM) [39] suggests that the impact on methanogens and protozoa is highly dependent on supplementation level, potentially compounded by differences in animal species (dairy cows vs. Simmental cattle) or dietary structure.

Tannins can inhibit fibrolytic activity by binding to microbial surface enzymes or directly complexing with cellulose, potentially suppressing the growth of fiber-degrading bacteria [40]. The primary fibrolytic consortium in rumen fluid comprises *Ruminococcus flavefaciens*, *Fibrobacter succinogenes*, and *Ruminococcus albus*, constituting approximately 2.6%, 33%, and 46% of this population, respectively [41,42]. In this study, dietary supplementation with tannins at 0.4% DM did not significantly alter the relative abundance of *F. succinogenes* or *R. flavefaciens*, but significantly reduced *R. albus*. This contrasts with findings by Li Dabiao et al. (reduction in all three bacteria at 2% and 6% tannins in sheep/goats) [43] and Jayanegara et al. (reduction in F. succinogenes and *R. flavefaciens* with quebracho tannins in vitro) [17], highlighting that the inhibitory effects on specific fibrolytic bacteria vary considerably with tannin type, dose, and host species. Despite significant methanogen inhibition (22.1–27.7%), total VFA concentration remained unchanged, reflecting metabolic compensatory mechanisms within the ruminal ecosystem. Methane suppression redirected metabolic hydrogen toward alternative electron sinks, primarily favoring propionate synthesis via the succinate pathway (propionate proportion increased 64.2%) [44]. The marked decrease in the acetate-to-propionate ratio (reduced 31.4%) further corroborated this shift. Critically, core fibrolytic populations—*Fibrobacter succinogenes*, *Butyrivibrio fibrisolvens*, and *Prevotella*—maintained stability, sustaining carbohydrate fermentation capacity and preserving total VFA output during the metabolic transition from methanogenesis to propionogenesis [45]. Crucially, despite the reduction in R. albus, the digestibility of NDF and ADF remained unaffected at the 0.4% DM supplementation level. This indicates that the decrease in *R. albus* abundance did not compromise fiber digestion in dairy cows under these conditions, likely due to functional redundancy within the fibrolytic community and the strong degradative capacity of *F. succinogenes* for structural carbohydrates. Given the high diversity of cellulolytic microorganisms, our analysis focused solely on dominant populations. We therefore hypothesize that unobserved microbial communities may have contributed critically through functional compensation [46].

Historically classified as antinutritional factors, tannins can impair intake and nutrient digestibility, primarily at supplementation levels exceeding 3–4% DM [47]. However, consistent with studies using low tannin doses (0.4–1.0% DM) in calves [48], the present study observed no adverse effects on DM, OM, CP, or NDF intake or digestibility at 0.4% DM supplementation. Notably, tannic acid alone significantly increased crude protein digestibility (6.6%), while tea polyphenols alone and their combination increased it by 5% and 3.7%, respectively. This contrasts with the typical paradigm where tannin–protein binding might reduce CP digestibility by limiting ruminal degradation, suggesting that low-dose supplementation or specific tannin sources may enhance nitrogen utilization efficiency [49]. Further supporting improved nitrogen metabolism, all tannin treatments significantly reduced ruminal ammonia-N (by 23% individually and 34.5% synergistically) and milk urea nitrogen (MUN) (by 18.75%, 16.4%, and 21.1%, respectively). This reduction in ammonia-N and MUN aligns with the primary mechanism of tannins forming reversible complexes with dietary protein, thereby reducing ruminal proteolysis and ammonia production [50], and increasing the flow of digestible protein to the small intestine. While studies report variable MUN responses (e.g., reduction at 1.8% DM CTE vs. no effect at 0.45–0.9% DM [51]), the significant decrease observed here at 0.4% DM, despite similar dietary CP and RDP:RUP ratios across treatments, indicates a direct positive impact of these tannins on nitrogen utilization efficiency independent of diet formulation.

The observed reduction in MUN has significant practical implications. As MUN exhibits a positive linear relationship with urinary nitrogen excretion [20], its decrease indicates reduced environmental nitrogen loss. Furthermore, elevated MUN typically signals dietary CP overfeeding [52]; its reduction here, coupled with significant increases in milk protein concentration (within the optimal 3.0–3.2% range [53]) for individual tannin and tea polyphenol supplements and a significant increase for their combination, demonstrates improved nitrogen partitioning towards milk synthesis rather than waste. This enhancement in milk protein output is consistent with the increased flow of amino acids (AA) to the small intestine resulting from tannin-mediated protection of dietary protein from ruminal degradation [13]. Consequently, the supplementation, particularly the synergistic combination of tannic acid and tea polyphenols at 0.4% DM, effectively reduces urinary nitrogen excretion while simultaneously enhancing the protein quality of milk.

Extensive in vitro [49] and in vivo [54] studies demonstrate that dietary tannins suppress ruminal methane (CH_4_) production. Consistent with this, all tannin treatments in the present study significantly reduced CH_4_ emissions without inter-group variation. This mitigation primarily stems from tannins directly inhibiting methanogenic archaea and indirectly limiting protozoal populations—key symbiotic partners of methanogens [38]—thereby disrupting methanogenesis. The observed reduction aligns with the well-established negative correlation between plant condensed tannin (CT) content and in situ degradability/in vitro fermentation parameters, including CH_4_ output [13]. Furthermore, the metabolic pathways of key rumen metabolites contribute to this effect: while acetogenesis releases H_2_ (a substrate for methanogenesis), propionate synthesis consumes H^+^/H_2_, creating an antimethanogenic sink [55]. Consequently, the decreased acetate-to-propionate ratio induced by tannins (as reported earlier) directly contributes to CH_4_ suppression. This mechanistic framework is corroborated by Tavendale et al., who proposed tannins reduce methanogenesis via archaeal inhibition or H_2_ limitation [56].

Although all tannin treatments significantly reduced the abundance of total methanogenic archaea—the primary CH_4_ producers [57]—the magnitude of archaeal suppression did not fully account for the observed CH_4_ reduction. This discrepancy implies that diminished H_2_ availability, mediated through alternative microbial pathways, plays a critical role. Rumen microbial communities dynamically regulate hydrogen partial pressure (a key determinant of methanogenesis), with H_2_ generated during fiber fermentation and primarily consumed by methanogens. Notably, genomic and transcriptomic analyses reveal that ~67% of rumen microbial genomes encode and express hydrogenotrophic enzymes [58], indicating substantial functional redundancy for H_2_ utilization beyond methanogenesis. Thermodynamic and kinetic models confirm H_2_ pressure dictates CH_4_ yield and fermentation balance [59]. In this context, tannin-induced reductions in Ruminococcus albus (across all treatments) and Ruminococcus flavefaciens (with tannic acid alone)—major H_2_-producing, cellulolytic bacteria [40]—likely curtailed H_2_ supply to methanogens, synergizing with direct archaeal inhibition to amplify CH_4_ suppression. Concurrently, key fiber-degrading bacteria including *Fibrobacter succinogenes*, *Butyrivibrio fibrisolvens*, and *Prevotella* maintained stable populations across all treatments. *Ruminobacter amylophilus* showed a trend toward increase in T+TP with no statistically significant difference. These findings confirm the observed methane reduction without compromising fiber digestibility. Moreover, T significantly increased the propionate proportion, whereas TP exerted a milder suppression on *Ruminococcus flavefaciens*. Existing studies [60,61] corroborate that plant polyphenols mitigate methane emissions by selectively modulating microbial communities (e.g., protecting fibrolytic bacteria while inhibiting methanogens). This implies that distinct phenolic compounds achieve analogous methane reduction through unique microbial pathways.

## 5. Conclusions

This study establishes tea polyphenols (TP) as a natural modulator of rumen function, demonstrating that low-dose supplementation (0.4% DM) alone significantly enhances nitrogen utilization efficiency, reducing ammonia-N and methane emissions while increasing milk protein yield. Critically, TP exhibits comparable efficacy to tannic acid (T) in key metrics including methane suppression (13.68% for T; 10.09% for TP), crude protein digestibility enhancement (7.02% for T; 5.37% for TP) and milk protein enhancement (5.74% for T; 4.10% for TP), while offering inherent advantages as a plant-derived, food-safe compound. The synergistic combination of T and TP (50:50) shows relevant improvements: it maximizes nitrogen conservation (34.5% ruminal ammonia-N reduction; 21.1% MUN decrease), outperforming either polyphenol alone and amplifies milk protein synthesis, and no detrimental effects were observed on the measured rumen fermentation characteristics. By integrating TP metabolic precision with TA targeted protein-binding capacity, this approach unlocks multifunctional gains unattainable by single additives, suggests that plant–polyphenol synergism represents a promising strategy for reducing enteric methane emissions in dairy cattle, contributing to climate-smart livestock production.

## Figures and Tables

**Table 1:** Composition and nutritional value of the basal diet.

Composition (%DM)		Nutritional Level	
Oatgrass	2.21	NE_L_ (MJ/Kg)	6.47
^1^ Concentrate	0.1	CP (g/kg)	17.07
Sugar beet granulate	2.66	CF (g/kg)	6.89
Fat powders	1.33	NDF (g/kg)	35.64
Soybean meal	3.85	ADF (g/kg)	19.96
Whole cotton seed	3.84	Ca (g/kg)	0.60
Alfalfa hay	17.02	P (g/kg)	0.43
Corn flak	23.02		
Brewer’s grains	2.45		
Whole corn silage	43.52		

Note: ^1^ Concentrate is provided by Tianjin Lianying Feed Co., Ltd., Tianjin, China. Contents per kg premix: 100 mg Co, 8500 mg Cu, 50,000 mg Fe, 30,000 mg Mn, 30,000 mg Zn, 300 mg I, and 300 mg Se.

**Table 2:** PCR primers for real-time PCR assay.

Target Species	Primer Sequence (5′)	GeneBank Accession No.	TE (°C)	Size (bp)
Total bacteria	F: CGGCAACGAGCGCAACCC R: CCATTGTAGCACGTGTGTAGCC	AY548787.1	60	147
Total anaerobic fungi	F: GAGGAAGTAAAAGTCGTAACAAGGTTTC R: CAAATTCACAAAGGGTAGGATGATT	GQ355327.1	57.5	120
Total protozoa	F: GCTTTCGWTGGTAGTGTATT R: CTTGCCCTCYAATCGTWCT	HM212038.1	59	234
Total methanogens	F: TTCGGTGGATCDCARAGRGC R: GBARGTCGWAWCCGTAGAATCC	GQ339873.1	60	160
*R. albus*	F: CCCTAAAAGCAGTCTTAGTTCG R: CCTCCTTGCGGTTAGAACA	CP002403.1	60	176
*R. flavefaciens*	F: ATTGTCCCAGTTCAGATTGC R: GGCGTCCTCATTGCTGTTAG	AB849343.1	60	173
*B. fibrisolvens*	F: ACCGCATAAGCGCACGGA R: CGGGTCCATCTTGTACCGATAAAT	HQ404372.1	61	65
*F. succinogenes*	F: GTTCGGAATTACTGGGCGTAAA R: CGCCTGCCCCTGAACTATC	AB275512.1	61	121
*R. amylophilus*	F: CTGGGGAGCTGCCTGAATG R: GCATCTGAATGCGACTGGTTG	MH708240.1	60	102
*P. ruminicola*	F: GAAAGTCGGATTAATGCTCTATGTTG R: CATCCTATAGCGGTAAACCTTTGG	LT975683.1	58.5	74

**Table 3:** Effect of diet added with tannic acid and tea polyphenols on rumen fermentation in dairy cows.

Items	C ^1^	T	TP	T+TP	*p*-Values
pH	5.79 ± 0.15 ^b^	6.12 ± 0.14 ^a^	6.22 ± 0.03 ^a^	6.30 ± 0.17 ^a^	0.001
NH_3_-N (mg/dL)	13.16 ± 1.39 ^a^	10.09 ± 0.82 ^b^	10.09 ± 1.56 ^b^	8.62 ± 0.5 ^b^	0.001
MCP (g/L)	0.65 ± 0.02 ^a^	0.45 ± 0.03 ^b^	0.44 ± 0.08 ^b^	0.35 ± 0.04 ^b^	0.003
Methane production (L)	445.83 ± 3.14 ^a^	384.86 ± 13.7 ^b^	397.26 ± 16.53 ^b^	395.02 ± 7.18 ^b^	0.020
Acetate (A, mmol/L)	65.99 ± 4.47	63.96 ± 3.08	60.51 ± 4.83	57.89 ± 7.44	0.196
Propionate (P, mmol/L)	20.42 ± 0.89 ^c^	33.52 ± 3.05 ^a^	26.73 ± 3.89 ^b^	28.92 ± 3.33 ^ab^	0.008
Isobutyrate (mmol/L)	0.63 ± 0.23	0.60 ± 0.07	0.56 ± 0.08	0.60 ± 0.08	0.804
Butyrate (mmol/L)	10.92 ± 2.5	13.36 ± 2.7	13.37 ± 1.71	10.9 ± 0.84	0.110
Isovalerate (mmol/L)	1.17 ± 0.51	1.35 ± 0.32	0.98 ± 0.14	1.08 ± 0.14	0.226
Valerate (mmol/L)	1.75 ± 0.68	2.27 ± 0.85	2.12 ± 0.61	1.96 ± 0.28	0.709
TVFA (mmol/L)	105.29 ± 15.14	115.07 ± 2.27	104.06 ± 7.00	100.89 ± 10.91	0.178
A/P	2.80 ± 0.63 ^a^	1.92 ± 0.2 ^b^	2.37 ± 0.49 ^b^	2.09 ± 0.42 ^b^	0.100

Note: ^a,b^ Mean values in the same row with different superscripts differ (*p* < 0.05). ^1^ C, without tannic acid and tea polyphenols; T, 0.4% tannic acid added; TP, 0.4% tea polyphenols added; T+TP, 0.2% tannic acid and 0.2% tea polyphenols added.

**Table 4:** Effect of diet added with tannic acid and tea polyphenols on nutrient apparent digestibility of dairy cows (%).

Items	C ^1^	T	TP	T+TP	*p*-Values
Dry matter (%)	68.6 ± 1.67	70.6 ± 3.28	69.1 ± 2.45	69.0 ± 1.34	0.092
Organic matter (%)	66.0 ± 2.80	68.1 ± 3.30	67.5 ± 2.64	67.1 ± 1.44	0.067
Crude protein (%)	72.7 ± 3.46 ^b^	77.8 ± 3.07 ^a^	76.6 ± 1.92 ^ab^	75.5 ± 1.68 ^ab^	0.041
Ether extract (%)	82.4 ± 4.40	82.1 ± 2.87	80.8 ± 2.57	79.2 ± 2.41	0.892
Neutral detergent fiber (%)	56.1 ± 7.28	54.4 ± 5.42	51.8 ± 4.82	53.7 ± 5.53	0.079
Acid detergent fiber (%)	46.3 ± 6.84	48.7 ± 4.43	46.3 ± 4.47	46.8 ± 4.37	0.220

Note: ^a,b^ Mean values in the same row with different superscripts differ (*p* < 0.05). ^1^ C, without tannic acid and tea polyphenols; T, 0.4% tannic acid added; TP, 0.4% tea polyphenols added; T+TP, 0.2% tannic acid and 0.2% tea polyphenols added.

**Table 5:** Effect of diet with added tannic acid and tea polyphenols on blood indicators in dairy cows.

Items	C ^1^	T	TP	T+TP	*p*-Values
Triglycerides (mmol/L)	1.37 ± 0.36	0.98 ± 0.58	1.06 ± 0.53	1.20 ± 0.32	0.837
Total cholesterol (mmol/L)	3.93 ± 0.62	4.82 ± 0.02	5.07 ± 0.27	4.32 ± 1.10	0.194
Plasma urea nitrogen (mmol/L)	6.93 ± 0.40 ^a^	5.83 ± 0.21 ^b^	5.11 ± 0.42 ^b^	5.10 ± 0.80 ^b^	0.028
Creatinine (μmol/L)	118 ± 6.36	64.7 ± 2.77	77.7 ± 3.46	57.5 ± 1.68	0.251
Aspartate aminotransferase (U/L)	22.9 ± 4.56	25.8 ± 3.62	16.4 ± 7.36	19.5 ± 8.83	0.611
Alanine aminotransferase (U/L)	27.9 ± 2.28	22.9 ± 11.62	30.3 ± 9.45	24.1 ± 3.58	0.465
Albumin (g/L)	45.2 ± 2.55	47.1 ± 5.72	48.4 ± 2.95	49.1 ± 8.56	0.072
Glutamylphthalide aminotransferase (U/L)	33.9 ± 4.60	43.4 ± 0.72	34.9 ± 3.02	29.8 ± 1.54	0.560
Total protein (g/L)	70.2 ± 2.76	73.4 ± 5.65	75.2 ± 1.84	74.1 ± 0.35	0.079
Globulin (g/L)	25.0 ± 3.32	26.3 ± 6.98	27.3 ± 3.78	25.1 ± 1.98	0.134

Note: ^a,b^ Mean values in the same row with different superscripts differ (*p* < 0.05). ^1^ C, without tannic acid and tea polyphenols; T, 0.4% tannic acid added; TP, 0.4% tea polyphenols added; T+TP, 0.2% tannic acid and 0.2% tea polyphenols added.

**Table 6:** Effects of diet with added tannic acid and tea polyphenols on milk yield and components.

Items	C ^1^	T	TP	T+TP	*p*-Values
Milk yield (kg)	38.6 ± 1.28	39.6 ± 5.56	39.4 ± 2.52	39.9 ± 3.56	0.157
FCM (kg/d)	36.5 ± 1.27	38.0 ± 1.92	38.4 ± 2.14	37.7 ± 2.10	0.089
Milk protein production (kg)	1.22 ± 0.05	1.29 ± 0.18	1.27 ± 0.09	1.29 ± 0.11	0.074
Milk fat (%)	3.63 ± 0.07	3.72 ± 0.17	3.82 ± 0.30	3.63 ± 0.14	0.464
Milk protein (%)	3.15 ± 0.03 ^b^	3.25 ± 0.02 ^a^	3.22 ± 0.05 ^a^	3.23 ± 0.02 ^a^	0.001
Lactose (%)	5.19 ± 0.22	5.23 ± 0.17	5.17 ± 0.35	5.00 ± 0.26	0.673
Non-fat milk solids (%)	9.62 ± 0.21	9.33 ± 0.29	9.53 ± 0.34	9.35 ± 0.35	0.437
Total solids (%)	13.3 ± 0.27	13.1 ± 0.39	13.4 ± 0.63	13.0 ± 0.36	0.638
Urea nitrogen in the milk (mg/dL)	12.8 ± 0.25 ^a^	10.4 ± 0.9 ^b^	10.7 ± 1.12 ^b^	10.1 ± 0.98 ^b^	0.022
Somatic cell count (10^4^/mL)	8.70 ± 10.02	8.41 ± 6.24	7.63 ± 3.79	7.82 ± 2.59	0.153

Note: ^a,b^ Mean values in the same row with different superscripts differ (*p* < 0.05). ^1^ C, without tannic acid and tea polyphenols; T, 0.4% tannic acid added; TP, 0.4% tea polyphenols added; T+TP, 0.2% tannic and 0.2% tea polyphenols added.

## Data Availability

The original contributions presented in this study are included in the article/Supplementary Material. Further inquiries can be directed to the corresponding author.

## Supplementary Materials

The following supporting information can be downloaded at: https://www.mdpi.com/article/10.3390/microorganisms13081848/s1, Figure S1: The standard curves obtained by plotting the logarithm of DNA concentration for Anaerobic (a), *B. fibrisolvens* (b), Prevotella (c), Total bacteria (d), *R. amylophilus* (e), Methanogens (f), *F. succinogenes* (g), *R. flavefaciens* (h), *R. albus* (i) and Protozoa (j) versus threshold cycle (Ct) for population quantification by using real time PCR.
