# Supplementary material for: Effects of Dietary Tannic Acid and Tea Polyphenol Supplementation on Rumen Fermentation, Methane Emissions, Milk Protein Synthesis and Microbiota in Cows
Source: Microorganisms. 2025 Aug 7;13(8):1848. doi: 10.3390/microorganisms13081848 (PMC12388258; doi:10.3390/microorganisms13081848)

Figure S1. The standard curves obtained by plotting the logarithm of DNA concentration for Anaerobic (a), *B.fibrisolvens* (b), Prevotella (c), Total bacteria (d), *R.amylophilus* (e), Methanogens (f), *F.succinogenes* (g), *R.flavefaciens* (h), *R.albus* (i) and Protozoa (j) versus threshold cycle (Ct) for population quantification by using real time PCR.

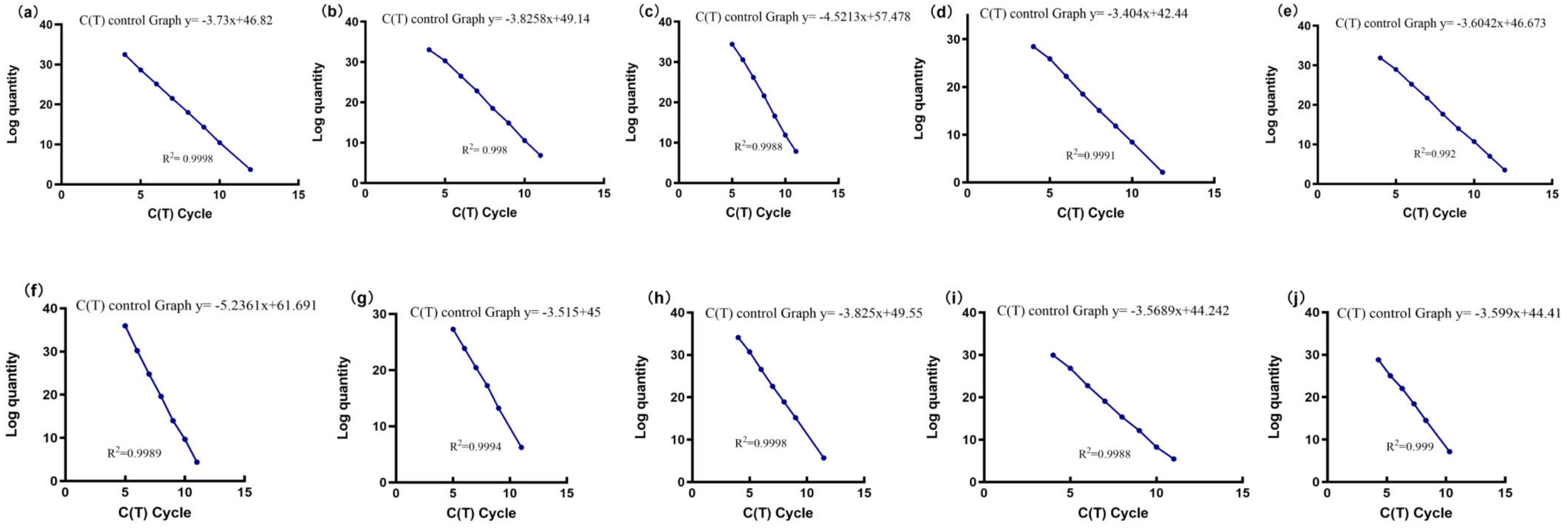

Supplement: Supplementary file 1 [file microorganisms-13-01848-s001.zip › microorganisms-3757148-supplementary.pdf]
